# Golden Rules of Surgical Practice

**Published:** 1892-03

**Authors:** 


*Golden Rules of Surgical Practice.* By A HOSPITAL SURGEON. Pp. 53. Bristol: JOHN WRIGHT & CO. [n.d.]

For the weak and the rash and the ignorant and the foolish, aphorisms and dicta have their value; but amongst the educated, in this age of culture, sound practice is best based on well-reasoned knowledge. We confess to a hearty dislike for all rules of thumb, even when written for "dressers and junior house surgeons," and this dainty little booklet in no degree removes this dislike.

There is little to cavil at as regards the sense of the rules; they are mostly common and trite enough, and therefore mostly correct. The author assumes perhaps too much, however, when he calls the rules "golden." These, for instance—"Never forget to warn your patient that a Colles' fracture, even when treated with the greatest care, leaves some deformity," "Never forget to warn the parents of a harelip *(sic!)* that one operation is usually inadequate"—suggest that the author's surgery is not of that sort from which golden rules can be readily evolved.

